# Early disruption of mitochondrial response to nutrients is a common feature in Alzheimer's disease

**DOI:** 10.1002/alz70855_097885

**Published:** 2025-12-23

**Authors:** Evelyn Pardo, Vijay Kumar Sagar, Kelly Kim, Ammasi Periasamy, George S Bloom, Andrés Norambuena

**Affiliations:** ^1^ University of Virginia, Charlottesville, VA, USA

## Abstract

**Background:**

Mitochondrial dysfunction, oxidative stress and mTOR dysregulation are defining features of Alzheimer's disease (AD). We discovered ‘Nutrient‐induced Mitochondrial Activity’ (NiMA), an inter‐organelle signaling pathway whereby insulin stimulation of lysosomal mTORC1 regulates mitochondrial activity and mtDNA synthesis in neurons in culture. We also reported NiMA to be downregulated by extracellular amyloid‐β oligomers (AβOs) in neuron cultures (DOI: 10.15252/embj.2018100241.) The mechanism involves AβO‐induced activation of mTORC1 at the neuronal plasma membrane (DOI: 10.1016/j.jalz.2016.08.015) and upregulation of superoxide dismutase 1 (SOD1), a major regulator of cellular redox (DOI: 10.1016/j.nbd.2022.105737). These observations raise the possibility that AβOs and AD risk factors known to inhibit insulin signaling disrupt NiMA at presymptomatic stages of AD. Here, we report that AβOs and APOE4 expression disrupt NiMA at presymptomatic AD stages and describe molecular mechanisms that are involved.

**Method:**

Live mitochondrial metabolism in brains of APP knock‐in (APPKI) mice harboring Swedish, Arctic and Austrian mutations, and of APOE4 knock‐in (APOE4KI) and APOE 3 (APOE3KI) mice were recorded with two‐photon fluorescence lifetime imaging.

**Result:**

We found NiMA to be downregulated in 4‐month‐old APPKI mice and completely blocked in 6‐month‐old animals. Disruption of NiMA, thus occurs ∼2‐3 months before microgliosis, cognitive decline and other pathological AD features detected in this Aβ mouse model. Mechanistically, we found that GSK3β signals through mTORC1 to regulate SOD1 and mitochondrial activity. Pharmacological inhibition of GSK3β in 4‐month‐old APPKI mice partially restored mitochondrial functioning, suggesting that GSK3β‐mediated regulation of NiMA controls SOD1 interaction with cytosolic regulators. BioID, a proximity‐dependent method for identifying protein‐protein interactions in living cells, identified 10 potential novel regulators of NiMA. Additionally, NiMA was downregulated in 2 month old APOE4 KI mice. The latter result represents the earliest molecular dysfunction reported for APOE4 expression in mice. Thus, we are unveiling a fundamental mechanism connecting nutrient sensing, mTORC1 kinase activity and cytosolic redox to mitochondrial functioning in neurons.

**Conclusion:**

Our results indicate that NiMA disruption is an early event in AD pathogenesis.

